# Structural Remodeling of Phage φPNJ‐6 Hoc Promotes Adhesion to the Intestinal Epithelium

**DOI:** 10.1155/tbed/2424208

**Published:** 2026-06-09

**Authors:** Linlin Ye, Xuhang Wang, Jiaqi Cui, Xinru Chen, Pan Tao, Yuepeng Liu, Yuhan Zhang, Feng Xue, Jianjun Dai, Fang Tang

**Affiliations:** ^1^ MOE Joint International Research Laboratory of Animal Health and Food Safety, Key Laboratory of Animal Bacteriology, Ministry of Agriculture, College of Veterinary Medicine, Nanjing Agricultural University, Nanjing, China, njau.edu.cn; ^2^ National Key Laboratory of Agricultural Microbiology, Hubei Hongshan Laboratory, Huazhong Agricultural University, Wuhan, 430070, China, hzau.edu.cn; ^3^ Xiangyang Academy of Agricultural Sciences, Xiangyang, China; ^4^ School of Life Science and Technology, China Pharmaceutical University, Nanjing, China, cpu.edu.cn

**Keywords:** adhesion, bacteriophage, CRISPR-Cas, *Escherichia coli*, Hoc protein, mucus

## Abstract

Although the mechanisms underlying bacteriophage‐host interactions have been extensively elucidated, the “nonlytic” interactions between bacteriophage and the host intestinal microenvironment remain an emerging field. Our previous work demonstrated that the *Escherichia coli* T4‐like bacteriophage φPNJ‐6 adheres to the mucin MUC2 via the capsid protein Hoc. However, whether a rigid lock‐and‐key model fully explains this interaction remains unclear. In this study, we employed CRISPR‐Cas12a‐mediated precise deletion to remove the key Hoc residues involved in MUC2 binding (aa29−33) from the φPNJ‐6 genome, generating the mutant strain Hoc^Δ29−33^‐PNJ‐6. Unexpectedly, both in vitro and in vivo experiments revealed that the engineered bacteriophage Hoc^Δ29−33^‐PNJ‐6 exhibited significantly enhanced adhesion. Structural analysis of Hoc showed that the deletion altered its conformation and increased the number of L‐fucose‐binding sites. These changes confer stronger fucose affinity to Hoc^Δ29−33^, thereby promoting intestinal colonization by φPNJ‐6. Overall, these results indicate that the Hoc‐MUC2 interaction does not conform to a simple lock‐and‐key model and that structural remodeling of Hoc significantly affects the adhesion capacity of φPNJ‐6. Our findings provide a strategy for designing long‐term mucosa‐resident therapeutic bacteriophages and expand the theoretical framework of bacteriophage–host microenvironment interactions.

## 1. Introduction

Bacteriophages (phages) are the most abundant and diverse biological entities on Earth and are found wherever bacteria exist [1–3]. In some ecosystems, phages outnumber bacteria by up to tenfold. Based on morphology, phages are generally classified into filamentous phages, tailed phages, and nontailed phages. Tailed phages include three families *Myoviridae*, which possess retractable tails, *Siphoviridae*, characterized by long, nonretractable tails, and *Podoviridae*, which have short tails. Among these groups, T phage and λ phage are the most extensively studied [4–7]. Phages are widely distributed across diverse biological habitats, including the skin and internal organs [8]. However, their highest abundance is found in the intestinal epithelial mucosal layer [9]. Studies indicate that ~90% of the viruses in the gastrointestinal tract, particularly within the gastrointestinal mucosa, are phages [10]. The gastrointestinal mucosal environment is highly complex, and the mucus layer serves as a protective barrier that maintains a dynamic equilibrium between eukaryotic cells and the intestinal microbiota [11–13]. The mucus layer is primarily composed of high‐molecular‐weight mucins, often exceeding 400 kDa, which are highly glycosylated and form a mesh‐like structure over the epithelial surface [14]. Mucins are encoded by specific mucin genes and are categorized into three main types secreted mucins, membrane‐bound mucins, and soluble mucins. Among them, the secreted mucin MUC2 is the predominant protein component of intestinal mucus. It is produced by goblet cells and is primarily located on the mucosal surface of the small and large intestines, protecting epithelial cells from digestive enzyme damage [15, 16]. In addition, the highly glycosylated mucins in the intestines serve as a nutrient source for intestinal bacteria, leading to a higher bacterial density than in the surrounding environment [17]. Together, the presence of the mucus layer and the high bacterial concentration create favorable conditions for phage colonization.

T4 phages are among the phage types with the largest genomes identified to date and consist of an icosahedral head capsid and a contractile tail. The head capsid is mainly composed of the major capsid protein gp23, which assembles into a hexagonal lattice, with 155 gp23 hexamers forming the structural framework of the phage head [18, 19]. The center of each gp23 hexamer serves as a binding site for the highly antigenic outer capsid protein Hoc [19]. Hoc is located on the surface of the T4 phage head and consists of a conserved C‐terminal capsid‐binding domain and three immunoglobulin (Ig)‐like domains [20]. Hoc is not essential for capsid assembly and associates with the phage capsid only during the final stage of virion maturation [21, 22]. With increasing insight into phage biology, it has become evident that the Hoc protein exposed on the capsid surface is associated with phage adhesion. Barr proposed that phages are more abundant in mucus‐rich environments than in surrounding nonmucosal environments, indicating an intrinsic affinity of phages for mucus. Based on this observation, their team proposed the Bacteriophage Adherence to mucus (BAM) model [23]. According to this model, phages adhere to and accumulate within the mucus layer through the interaction between specific protein domains displayed on the phage capsid surface and the glycan chains of mucins in the mucus. Our team previously investigated the adhesion capacity of *Escherichia coli* phage φPNJ‐6, a T4 phage with Hoc on its head capsid. The host strain, *E. coli* CVCC232, is an enterotoxigenic *E. coli* (ETEC) that expresses K99 (F5) fimbriae, a key virulence factor responsible for diarrhea in young livestock. Therefore, elucidating how the Hoc protein influences phage adhesion could inform the development of strategies to prevent or treat infections caused by this pathogen. Through point mutation and antibody blocking experiments, we identified Hoc as the key protein mediating the adhesion of φPNJ‐6 to intestinal mucosa. Specifically, Hoc interacts with fucose residues of the MUC2 protein in the mucus layer via amino acids 29–33, thereby facilitating phage targeting of pathogenic bacteria [24]. Based on these findings, we aim to further investigate whether the “lock‐and‐key” model is sufficient to explain the interaction between Hoc and MUC2.

In this study, to investigate the role of the Hoc protein in phage adhesion, we used the CRISPR‐Cas12a system to edit the genome of *E. coli* phage φPNJ‐6. This generated two mutant phages: ΔHoc‐PNJ‐6, which lacks the entire Hoc protein, and Hoc^Δ29−33^‐PNJ‐6, which lacks the key binding sites of Hoc. In both in vivo and in vitro experiments, Hoc^Δ29−33^ retained the ability to bind MUC2, and the phage Hoc^Δ29−33^‐PNJ‐6 exhibited enhanced intestinal colonization. Further structural analysis of Hoc revealed that deletion of amino acids 29–33 induced conformational changes in the protein, increasing the number of fucose‐binding sites and enhancing its affinity for fucose, thereby promoting phage colonization in the intestine. These findings indicate that the interaction between Hoc and MUC2 is not governed by a simple “lock‐and‐key” model and that structural changes in Hoc directly modulate the adhesion capacity of φPNJ‐6.

## 2. Materials and Methods

### 2.1. Strains, Phage, and Plasmids

The *E. coli* strain CVCC232 and phage φPNJ‐6 used in this study were previously isolated and preserved in our laboratory. DH5α competent cells (TSC‐C01, China) and BL21(DE3)‐competent cells (TSC‐E01, China) were purchased from Tsingke Biotechnology Co. Ltd. (Nanjing, China). The prokaryotic expression vector, pET‐28a, was used for protein expression. The LbCPF1 plasmid vector was kindly provided by Professor Tao Pan of Huazhong Agricultural University.

### 2.2. Generation and Screening of Phage Mutants via CRISPR‐Cas12a Editing

To generate the Hoc‐specific mutant phage, the LbCPF1 plasmid (Cas12a‐Δ29−33) and its corresponding donor plasmid (pET28a‐Δ29−33) were cotransformed into *E. coli* CVCC232. A control strain was generated by transforming CVCC232 with the Cas12a‐Δ29−33 plasmid alone. For plaque assays, transformed bacteria were mixed with wild‐type φPNJ‐6 at a 1:1 ratio, incubated at 37°C for 10 min, and subsequently mixed with 5 mL of semisolid agar before being overlaid onto LB agar plates. In the double‐layer agar assay, the bottom layer consists of LB solid medium containing 1.5% agar, while the overlay comprises a semisolid medium containing 0.5% agar. Following overnight incubation at 30°C, individual plaques were selected and subjected to three rounds of plaque purification to ensure clonality. Cas12a‐ΔHoc and pET28a‐ΔHoc plasmids were used to repeat the same process to obtain ΔHoc‐PNJ‐6 mutants. For sequence verification, a single purified plaque was transferred to 1 mL of SM buffer. A 4 μL aliquot of the supernatant served as a template for PCR amplification. The resulting PCR products were purified and sequenced by Tsingke Biotechnology Co., Ltd. (Nanjing, China). To assess the genetic stability of the engineered phage, 10 serial passages were conducted using sequence‐verified phage stocks. About 200 μL of the engineered phage and 200 μL of the host bacterium CVCC232 were combined in 5 mL of LB broth and incubated at 37°C with continuous shaking at 180 rpm for 3–5 h. Following incubation, the culture was clarified by passage through a 0.22 μm membrane filter, and the resultant filtrate was retained for subsequent passage. This procedure was repeated for a total of 10 consecutive passages. Upon completion of the final passage, phage DNA from the 10th passage was used as a PCR template for amplification and sequencing to verify the retention of the target gene.

### 2.3. Adhesion of Phage to Different Intestinal Cell Lines

HT‐29 and Caco‐2 cells were cultured in 12‐well plates. After the cells grew to a monolayer, the medium was discarded, and the cell monolayer was gently washed twice with PBS. Subsequently, serum‐free DMEM and engineered phage suspension (500 μL per well) were added. For the control group, 500 μL of the wild‐type φPNJ‐6 suspension was used. The plates were gently agitated to ensure mixing and incubated at 37°C for 2 h to allow phage adhesion. After incubation, the supernatant was carefully removed. Adherent cells were scraped using a cell scraper and resuspended. The phage load of the collected cell suspension was determined using the double‐layer agar plate method.

### 2.4. In Vitro Infection Assay

HT‐29 cells were cultured in 12‐well plates until a confluent monolayer was obtained. For the phage treatment group, phage suspension (10^9^ PFU/mL, 500 μL per well) was added, and the plates were incubated at 37°C with 5% CO_2_ for 30 min. Following incubation, the cells were gently washed twice with PBS to remove the nonadherent phage. Subsequently, DMEM and bacterial suspension (10^8^ CFU/mL, 500 μL per well) were added to each well. In the negative control group, an equivalent volume of PBS was used instead of the bacterial suspension. Samples were collected at 3, 6, and 12 h postinfection for analysis. At each time point, 1 mL of PBS was gently added to each well, and the cells were scraped and transferred to microcentrifuge tubes. Then, 10 μL of the cell suspension was collected to measure the number of living cells using an automatic cell counter. The remaining sample was subjected to quantitative analysis to determine the counts of both bacteria and phages adherent to the cells.

### 2.5. Protein Structural Prediction and Fucose Docking Analysis

The amino acid sequences of the wild‐type Hoc protein and the Hoc^Δ29−33^ mutant were converted into the FASTA format using SnapGene software. Sequences for Hoc and Hoc^Δ29−33^ are provided in Supporting Information 1: Table S1. The FASTA‐formatted sequences were submitted to the I‐TASSER server (https://zhanggroup.org/) for protein structure prediction. The predicted model with the highest C‐score and a TM‐score greater than 0.5 was selected for each protein, ensuring correct topology. The online tool POCASA was employed to predict potential binding pockets within the three‐dimensional structures of both Hoc and Hoc^Δ29−33^. Subsequently, the predicted protein structures were uploaded to the Glycan Docking server (https://www.glycaninsight.cn/index/) to predict potential binding sites for small molecules. The final protein structures and their docking poses with L‐fucose were visualized and analyzed using the PyMOL molecular graphics system.

### 2.6. In Vitro Immunofluorescence Assay

HT‐29 cells were seeded into 15 mm glass‐bottom culture dishes (Nest Biotechnology, China) and cultured until a confluent monolayer was formed. A mixture of 100 μL protein solution and 100 μL phenol red‐free medium was added to the center of each culture dish and incubated for 2 h. Cells were fixed with 4% paraformaldehyde for 30 min and washed thrice with PBS. Nonspecific binding sites were blocked with 3% bovine serum albumin (BSA) for 1 h, after which the cells were washed three times with PBS. The primary antibody, diluted 1:200 in PBS, was applied and incubated at room temperature for 1.5 h. Following three washes with PBS, the samples were incubated with a 1:500 dilution of the fluorescently labeled secondary antibody for 45 min at room temperature in the dark. After three additional PBS washes, the nuclei were counterstained with 200 μL DAPI (Hoechst 33342 Stain Solution, Solarbio, China) for 5 min at room temperature. Finally, the cells were washed three times with PBS and maintained in 1 mL of PBS for imaging. Fluorescence images were captured using an inverted fluorescence microscope.

### 2.7. Co‐Immunoprecipitation (Co‐IP) Assay

Co‐IP assays were performed to evaluate the interaction between wild‐type Hoc/Hoc^Δ29−33^ proteins and MUC2. The MUC2 antibody was first incubated with protein A/G magnetic beads for 2 h at 4°C with gentle rotation. Subsequently, cell lysate was added to the bead–antibody complex and incubated for 8 h at 4°C. Following this incubation, either wild‐type Hoc or Hoc^Δ29−33^ protein was added to the respective mixtures and incubated overnight at 4°C. The final immunoprecipitates were washed extensively and prepared as samples for western blot analysis to detect potential protein–protein interactions.

### 2.8. Surface Plasmon Resonance (SPR) Assay

The carboxymethylated dextran matrix of the CM5 sensor chip was activated with a 1:1 mixture of 400 mM EDC (N‐ethyl‐N′(dimethylaminopropyl)carbodiimide) and 100 mM NHS (N‐hydroxysuccinimide) at a flow rate of 10 μL/min. Recombinant Hoc proteins were immobilized onto the activated chip surface as ligands using standard amine coupling chemistry. Following protein immobilization, the remaining activated groups were deactivated with 1 M ethanolamine hydrochloride (pH 8.5) for 7 min. L‐Fucose was serially diluted in running buffer (1 × PBS, 1% DMSO, and 0.005% Tween 20) to generate a concentration gradient ranging from 0.20 to 13 μM. The analyte solutions were injected over the protein‐immobilized flow cells at a flow rate of 20 μL/min for 100 s, followed by a 180 s dissociation phase. The binding cycle consisted of seven consecutive injections in an ascending order of concentration. Regeneration of the chip surface was performed between each binding cycle using 10 mM glycine‐HCl (pH 2.0) with a 30 s injection. All experiments were conducted at 25°C using a Biacore T200 system (Cytiva).

### 2.9. Determination of Phage Adhesion Capacity In Vivo

Four‐week‐old female BALB/c mice were randomly assigned to four experimental groups (*n* = 3). Before the experiment, streptomycin sulfate (5 g/L) was administered in drinking water for 24 h to reduce the resident intestinal microbiota, followed by a 24 h fasting period with water deprivation. Mice were then intragastrically administered 200 μL of a 5% sodium bicarbonate solution to neutralize gastric acidity. After 30 min, the mice received 200 μL of either wild‐type φPNJ‐6, ΔHoc‐PNJ‐6, Hoc^Δ29−33^‐PNJ‐6 (10^9^ PFU/mL), or PBS (control group) via intragastric gavage. Given that CVCC232 primarily colonizes and proliferates in the large intestinal segment of mice, and considering that this region possesses a thicker, more structurally defined mucus layer, which provides a unique and stable microenvironment for bacterium‐phage interactions [25], we collected samples from the cecum, colon, and feces at specified time points (12, 24, and 30 h) postadministration. The tissue samples were homogenized in PBS, and phage titers in the homogenates were subsequently determined using the double agar overlay plaque assay.

### 2.10. Mouse Prevention Experiment

Mice were randomly divided into four groups (*n* = 3). The pretreatment procedure was identical to that described in the “Determination of phage adhesion capacity in vivo” section. After 30 min, the mice received 200 μL of either wild‐type φPNJ‐6, ΔHoc‐PNJ‐6, Hoc^Δ29−33^‐PNJ‐6 (10^9^ PFU/mL), or PBS (control) via intragastric gavage. All mice were challenged with 200 μL *E. coli* CVCC232 suspension (10^9^ CFU/mL) at 6 h after administration, and the cecum and colon of mice were collected at 12 h after challenge. The intestinal mucosa and luminal contents from the cecum and colon were collected separately. The phage and bacterial loads in these samples were quantified using the double agar overlay plaque assay and colony counting, respectively.

### 2.11. In Vivo Immunofluorescent Staining

Four‐week‐old female BALB/c mice were randomly assigned to four groups (*n* = 3) and subjected to the identical pretreatment regimen described above. The four groups were intragastrically administered 200 μL of wild‐type φPNJ‐6, ΔHoc‐PNJ‐6, Hoc^Δ29−33^‐PNJ‐6 (10^9^ PFU/mL), or PBS (control). Mice were euthanized, and colonic tissues were immediately collected 6 h after phage gavage. The tissues were fixed in 4% paraformaldehyde, embedded in paraffin, and sectioned into 5 μm thick slices. The sections were then subjected to immunofluorescence staining for subsequent microscopic analysis.

## 3. Results

### 3.1. Genetic Engineering of *E. coli* Phage φPNJ‐6

**Figure 1:**
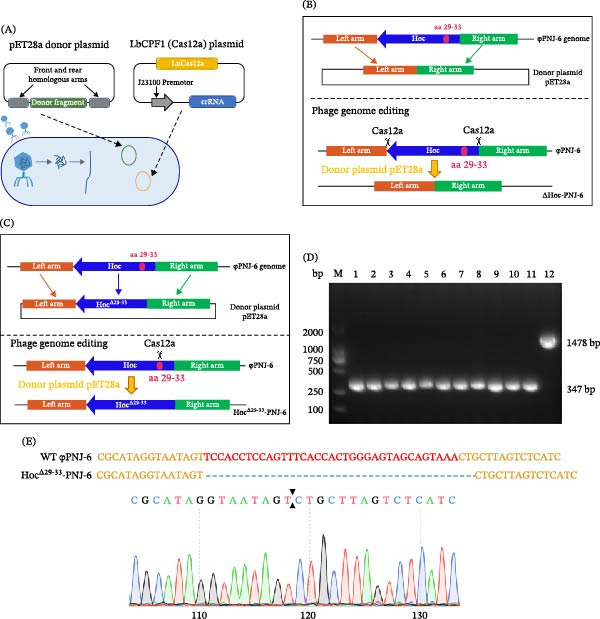
Phage modification. (A) Schematic diagram of plasmid construction and transformation. (B) Schematic diagram of the knockout strategy for the gene sequence encoding the Hoc protein. The upper part shows the donor fragment in the plasmid pET28a‐ΔHoc, and the lower part illustrates the strategy for deleting the *hoc* gene from the WT φPNJ‐6 genome. The genes flanking the *hoc* gene region are indicated. (C) Schematic diagram of the knockout strategy for the gene sequence encoding Hoc amino acids 29–33. The upper part shows the donor fragment in the plasmid pET28a‐Δ29−33, and the lower part illustrates the strategy for deleting the Hoc amino acids 29–33 from the WT φPNJ‐6 genome. The genes flanking the Hoc amino acids 29–33 region are indicated. (D) PCR identification of ΔHoc‐PNJ‐6 phage. The *hoc* gene is deleted. M: 2000 DNA marker; 1–11: ΔHoc‐PNJ‐6; 12: φPNJ‐6. (E) PCR identification peak chart of Hoc^Δ29−33^‐PNJ‐6. The sequencing results of the recombinant phage Hoc^Δ29−33^‐PNJ‐6 show that the *hoc* gene was replaced by the *hoc* ^Δ*29−33*^ gene.

In our previous studies, we found that the capsid protein Hoc of *E. coli* phage φPNJ‐6 is closely associated with its ability to adhere to the intestinal mucosa. Point mutation experiments further identified the interaction site between Hoc and the intestinal mucin MUC2, localizing it to amino acids 29–33 of Hoc and the fucose residues at the O‐glycosylation terminus of MUC2 [24]. To investigate the function of this motif, we genetically modified φPNJ‐6 using the CRISPR‐Cas technology. The CRISPR‐Cas12a system and a donor plasmid were introduced into the host strain CVCC232 of φPNJ‐6 (Figure 1A), and recombinant strains were obtained. Wild‐type φPNJ‐6 was were cocultured with the recombinant host strain. Through CRISPR‐Cas12a and donor‐plasmid‐mediated homologous recombination, we generated ΔHoc‐PNJ‐6, which lacks the entire Hoc protein (Figure 1B), and Hoc^Δ29−33^‐PNJ‐6, which lacks amino acids 29–33 of Hoc (Figure 1C). PCR analysis confirmed the successful construction of both recombinant phages (Figure 1D,E). Following 10 consecutive passages, PCR analysis confirmed that the engineered phage exhibited excellent genetic stability. The target deletion fragment remained stable after multiple passages, and no reversion mutations or fragment loss was detected (Supporting Information 2: Figure S1).

### 3.2. Characterization of the Biological Properties of Engineered Phage

Although deletion of the *hoc* gene does not impair the ability of the phage to complete its replication cycle, previous studies have shown that removal of nonessential phage genes can affect structural stability and lytic capacity [26]. Therefore, we systematically evaluated the impact of complete Hoc deletion or key epitope deletion on the biological properties and lytic ability of φPNJ‐6.

**Figure 2:**
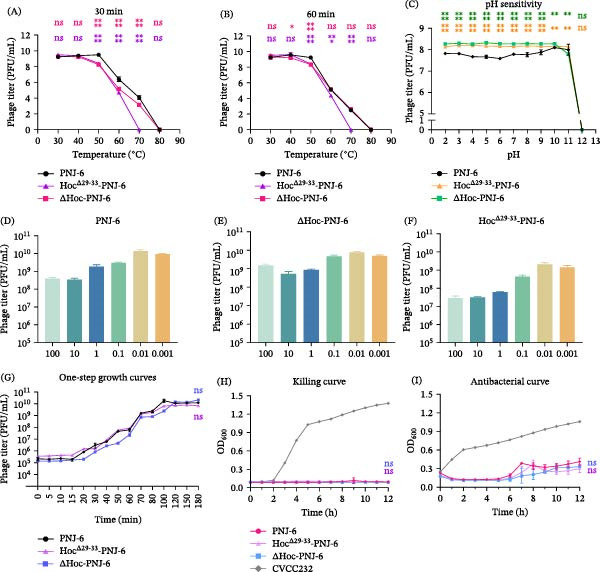
Comparison of the biological properties and lytic capacity of phage φPNJ‐6, Hoc^Δ29−33^‐PNJ‐6, and ΔHoc‐PNJ‐6. (A,B) Comparison of the thermal stability of the modified phage. (C) Comparison of acid–base sensitivity of modified phage. (D−F) The optimal multiplicity of infection of φPNJ‐6 (D), Hoc^Δ29−33^‐PNJ‐6 (E), and ΔHoc‐PNJ‐6 (F). (G) Comparison of one‐step growth curves of modified phage. (H,I) Comparison of the lysis ability of the modified phage, including the bactericidal curve (H) and the bacteriostatic curve (I).  ^∗^ *p* < 0.05;  ^∗∗^ *p* < 0.01;  ^∗∗∗^ *p* < 0.001;  ^∗∗∗∗^ *p* < 0.0001.

To test phage thermal stability, the phages were incubated at temperatures ranging from 30 to 80°C for specified periods, and the resulting phage titers were measured. The results showed that all three phages remained stable between 30 and 50°C (Figure 2A,B). When the temperature was increased to 60°C, the titers of all three phages decreased by four orders of magnitude. After 30 min at 70°C, Hoc^Δ29−33^‐PNJ‐6 was completely inactivated, while φPNJ‐6 and ΔHoc‐PNJ‐6 were fully inactivated after 30 min at 80°C (Figure 2A,B). These results indicate that the thermal tolerance of ΔHoc‐PNJ‐6 is comparable to that of the wild‐type phage, whereas Hoc^Δ29−33^‐PNJ‐6 exhibits reduced heat resistance, suggesting a decrease in its thermal stability.

Environmental pH is considered an important factor affecting phage stability. The phages were exposed to environments with pH values ranging from 2 to 12, and the resulting phage titers were measured. All three phages exhibited good adaptability within the pH range of 2–11 but were completely inactivated at pH 12 (Figure 2C), indicating intolerance to strongly alkaline conditions. When the pH was below 10, Hoc^Δ29−33^‐PNJ‐6 and ΔHoc‐PNJ‐6 exhibited slightly higher tolerance to acidity and alkaline conditions than φPNJ‐6, although the difference was less than one order of magnitude (Figure 2C). These results suggest that, compared to temperature, environmental pH has a smaller effect on the stability of the three phages, and the genetic modifications did not substantially alter their pH sensitivity.

The multiplicity of infection (MOI), defined as the ratio of phage particles to host cells [27], was determined, and the optimal MOI for all three phages was 0.01 (Figure 2D−F). Based on the optimal MOI, one‐step growth curves were determined for each phage (Figure 2G). The latent period of φPNJ‐6 and Hoc^Δ29−33^‐PNJ‐6 was 15 min, with a logarithmic growth phase of 100 min, whereas ΔHoc‐PNJ‐6 exhibited a longer extended latent period of 20 min and an extended logarithmic phase of 120 min (Figure 2G). These results indicate that deletion of Hoc prolongs the latent period and delays the onset of the logarithmic growth phase.

The lytic capacities of the three phages were further evaluated using bactericidal assays at CFU ~10^5^ (Figure 2H) and bacterial growth inhibition assays at CFU ~10^8^ (Figure 2I). The results showed that both wild‐type and engineered phages exhibited strong bactericidal activity, maintaining OD_600_ values below 0.2 within 12 h at low bacterial density (Figure 2H). At high bacterial density, all three phages displayed comparable inhibitory effects for up to 6 h (Figure 2I). To assess lytic activity under acidic conditions mimicking the gastrointestinal environment, PNJ‐6, ΔHoc‐PNJ‐6, and Hoc^Δ29−33^‐PNJ‐6 were treated with SM buffer at pH 2.0, 4.0, and 6.0. Following treatment, the lytic activity of both engineered phages was not significantly different from that of PNJ‐6. This indicated that deletion or mutation of the Hoc protein did not significantly affect the lytic activity of the phages under the same pH conditions (Supporting Information 2: Figure S2). Collectively, these results indicate that the phage engineering strategies employed in this study did not significantly affect the phage lytic capacity in vitro.

According to the above results, environmental temperature had a significant impact on phage stability, with Hoc^Δ29−33^‐PNJ‐6 displaying reduced tolerance to high temperatures. Other than temperature sensitivity, these modifications did not significantly affect the biological properties or lytic capacity of φPNJ‐6.

### 3.3. Enhanced Cellular Adhesion and Protective Efficacy of Hoc^Δ29−33^‐PNJ‐6

**Figure 3:**
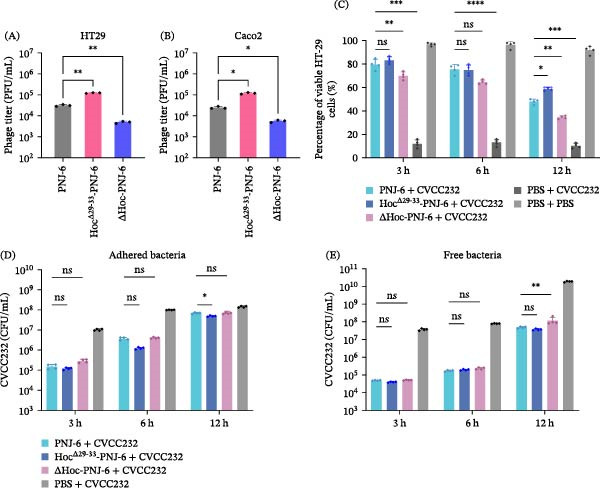
Adhesion capacity and protective efficacy of engineered phage on cultured cells. (A and B) Adhesion capacity of phage to HT‐29 (A) and Caco‐2 (B) cells. Phage titers associated with the cell monolayer were quantified after removal of the culture medium. (C) Protective efficacy of engineered phage on HT‐29 cell viability in the presence of CVCC232. (D,E) Bactericidal activity of engineered phage against host bacteria CVCC232, assessed by quantifying bacterial loads adherent to cells (D) and present in the planktonic phase of the culture supernatant (E).  ^∗^ *p* < 0.05;  ^∗∗^ *p* < 0.01;  ^∗∗∗^ *p* < 0.001;  ^∗∗∗∗^ *p* < 0.0001.

To assess the impact of genetic modifications on phage adhesion, we performed in vitro experiments using HT‐29 and Caco‐2 cell lines, both of which secrete mucin MUC2. After coincubation of the phage with the cells for 2 h, the phage titers adhering to the cells were measured using the double‐layer plaque assay. Using the φPNJ‐6 group as the control, ΔHoc‐PNJ‐6 showed a 1‐log reduction in adhesion compared to φPNJ‐6 in both HT‐29 (Figure 3A) and Caco‐2 (Figure 3B). This reduced adhesion demonstrated that the absence of the Hoc protein diminished the adhesion capacity of φPNJ‐6, consistent with our previous findings. Interestingly, Hoc^Δ29−33^‐PNJ‐6 displayed higher adhesion than φPNJ‐6, showing a 0.5–1 log increase in adhesion (Figure 3A,B).

Enhanced phage adhesion to the cell surface facilitates direct contact with pathogenic bacteria, thereby providing better protection to cells. To test this hypothesis, we performed phage‐bacteria–cell interaction experiments. Phage‐pretreated cells were coincubated with *E. coli* CVCC232. In the positive control group, both phages and bacteria were replaced with an equal volume of PBS, and in the negative control group, phage pretreatment was replaced with PBS. Cell culture medium and cells were collected at 3, 6, and 12 h postincubation to measure bacterial and phage loads, and live cell counts were conducted using a cell counter. Phage titer measurements showed no significant differences among the three phages under either adhesion or free conditions (Supporting Information 2: Figure S3). We propose that within the dynamic intestinal environment, in situ phage proliferation may have masked colonization differences attributable to variations in adhesion capability. Live cell counts revealed that, compared with φPNJ‐6, ΔHoc‐PNJ‐6 conferred lower cell protection, while Hoc^Δ29−33^‐PNJ‐6 showed enhanced cell protection (Figure 3C). At 12 h, the cell protection rate was 58.75% for the Hoc^Δ29−33^‐PNJ‐6 group, significantly higher than the 48% for φPNJ‐6 and 34.5% for ΔHoc‐PNJ‐6 (Figure 3C). These findings indicate that the reduced adhesion capacity of φPNJ‐6 due to Hoc protein deletion leads to a decreased cell protection ability, indirectly confirming the importance of Hoc protein for phage adhesion. Compared with the φPNJ‐6 and ΔHoc‐PNJ‐6, cells treated with Hoc^Δ29−33^‐PNJ‐6 exhibited reduced counts of both free and adhered bacteria (Figure 3D,E). Importantly, this effect was observed even when overall phage adhesion levels were not significantly different (Supporting Information 2: Figure S11–3), indicating that the improved colonization ability of Hoc^Δ29−33^‐PNJ‐6 promotes more effective bacterial clearance in vitro. These results indicate that deletion of the Hoc protein reduces φPNJ‐6 adhesion, while deletion of aa29−33 increases phage adhesion and facilitates bacterial elimination in the microenvironment.

### 3.4. Structural Prediction and Docking Analysis of Hoc and Hoc^Δ29−33^

**Figure 4:**
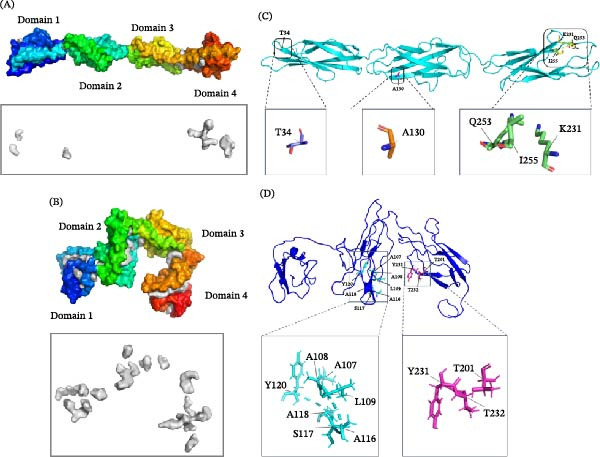
Structural prediction and fucose docking analysis of Hoc proteins. (A) Predicted structure of wild‐type Hoc protein, showing four distinct domains and five putative binding pockets. (B) Predicted structure of Hoc^Δ29−33^ mutant, maintaining the four‐domain architecture but containing 16 putative binding pockets. (C) Molecular docking visualization of L–fucose interaction sites on wild‐type Hoc, revealing five potential binding sites, including one adjacent to the aa29−33 region (T34). (D) Docking analysis of Hoc^Δ29−33^ with L‐fucose, identifying 10 potential interaction sites. All structural visualizations were generated using PyMOL Molecular Graphics System.

Based on the above results, we hypothesized that the deletion of aa29−33 in the Hoc protein may induce a conformational change, thereby altering its interaction with the mucosal environment. To validate this hypothesis, we performed structural predictions for both the Hoc and Hoc^Δ29−33^ proteins. Both proteins were predicted to contain four domains, with the first three corresponding to Ig‐like domains (Figure 4A,B). RMSD is a key metric for assessing the similarity of protein three‐dimensional structures. Structural comparison revealed that the RMSD between the two proteins was 3 Å, indicating significant overall structural changes and the emergence of clear conformational differences. Protein structure changes are often accompanied by alterations in protein pockets. Prediction of protein pockets showed that Hoc contained five pockets, whereas Hoc^Δ29−33^ contained 16 pockets (Figure 4A,B). This increase in the pocket number in Hoc^Δ29−33^ on Hoc^Δ29−33^‐PNJ‐6 may enhance its affinity for specific ligands, potentially explaining the increased adhesion capacity of Hoc^Δ29−33^‐PNJ‐6.

Our previous study has demonstrated that fucose residues serve as interaction sites between MUC2 and Hoc [24]. Therefore, we predicted the L‐fucose‐binding sites in Hoc and Hoc^Δ29−33^. A site with a probability greater than 0.5 was considered a potential fucose‐binding site. The results showed that the Ig‐like region of Hoc was predicted to contain three potential L‐fucose binding pockets, with a total of five possible binding sites (Figure 4C). Among these, only T34 in domain 1 corresponded to the interaction site identified in our previous study [24], whereas the other two predicted fucose‐binding pockets did not actually bind fucose. This indicates that not all predicted fucose‐binding pockets function in native interaction environments. Prediction of the Ig‐like region of Hoc^Δ29−33^ showed two potential L‐fucose binding pockets, containing a total of 10 possible binding sites, none of which overlapped with the L‐fucose‐binding pockets of Hoc (Figure 4D). Since not all predicted fucose‐binding pockets were functional, we did not focus on the decrease in the number of fucose‐binding pockets in Hoc^Δ29−33^. However, the observed increase in the number of L‐fucose binding sites in Hoc^Δ29−33^ suggests a potential correlation between the number of binding sites and the affinity of the protein for fucose.

### 3.5. Analysis of Binding Affinity Between Hoc/Hoc^Δ29−33^ and MUC2

**Figure 5:**
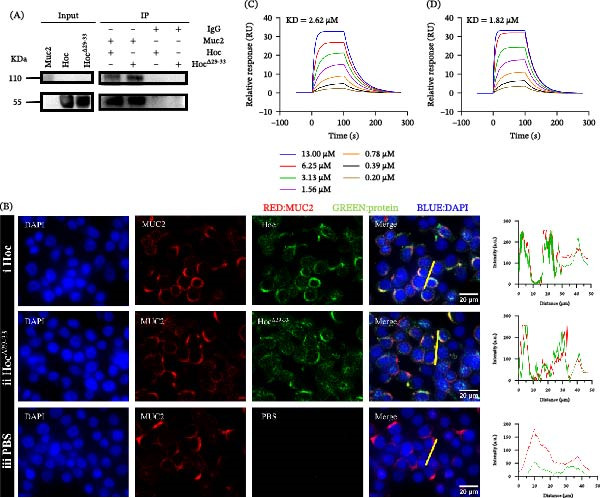
Analysis of binding affinity between Hoc/Hoc^Δ29−33^ and MUC2. (A) Co‐immunoprecipitation (Co‐IP) assays demonstrate the interaction between MUC2 and both wild‐type Hoc and Hoc^Δ29−33^ proteins. (B) Fluorescence colocalization imaging of Hoc proteins with intestinal cells. Red: MUC2; Blue: DAPI (nuclei); Green: Hoc proteins. Scale bar: 20 μm. Images are representative of three independent experiments. (C) Surface plasmon resonance (SPR) sensorgrams showing the binding kinetics of wild‐type Hoc protein to L‐fucose. (D) SPR analysis of the binding kinetics between Hoc^Δ29−33^ and L‐fucose.

The above experimental results suggest that our modification of Hoc may have induced structural changes, which, in turn, could alter its binding to MUC2. Therefore, we expressed and purified both Hoc and Hoc^Δ29−33^. The CO‐IP assay showed that Hoc^Δ29−33^ retained the ability to bind MUC2 (Figure 5A). To directly observe the interaction between Hoc, Hoc^Δ29−33^, and MUC2, we performed indirect immunofluorescence. Hoc^Δ29−33^ was co‐incubated with HT29 cells, with Hoc serving as the positive control and PBS as the negative control. Compared with the PBS group, both the Hoc and Hoc^Δ29−33^ groups exhibited significant colocalization with MUC2, with Hoc^Δ29−33^ showing a closer fit in the colocalization curve (Figure 5B). These results indicated that Hoc^Δ29−33^ retained the ability to bind MUC2.

MUC2 primarily interacts with Hoc via the fucose residues at the terminal positions of its O‐glycan chains [24]. To quantify this interaction, we employed SPR to measure the binding kinetics of Hoc, Hoc^Δ29−33^, and fucose. Hoc and Hoc^Δ29−33^ were immobilized on the sensor chip, and a fucose solution was injected at a constant flow rate to monitor association and dissociation. The results showed that the association rate (ka) of Hoc^Δ29−33^ was higher than that of Hoc, indicating that Hoc^Δ29−33^ had a higher initial binding efficiency with fucose. The dissociation rate (kd) of Hoc^Δ29−33^ was greater than that of Hoc, suggesting that the Hoc–fucose complex was more stable. The equilibrium dissociation constants (KD) were 2.62 μM for Hoc and 1.82 μM for Hoc^Δ29−33^, indicating that Hoc^Δ29−33^ exhibited overall stronger binding to fucose residues (Figure 5C,D). In conclusion, deletion of aa29−33 in Hoc^Δ29−33^ enhanced its binding to fucose, thereby increasing its affinity for MUC2.

### 3.6. Enhanced Gut Adhesion and Protective Efficacy of Hoc^Δ29−33^‐PNJ‐6 in Mice

To evaluate the changes in the adhesion ability of the engineered phage, we performed in vivo adhesion assays using a mouse model. Four‐week‐old mice were gavaged with φPNJ‐6, ΔHoc‐PNJ‐6, or Hoc^Δ29−33^‐PNJ‐6 in the absence of the host strain CVCC232. Phage retention in the intestinal mucosa and feces was measured at different time points by collecting intestinal and fecal samples. The control group received an equal amount of PBS. The results showed that all phages persisted in the mice for up to 30 h (Figure 6A−C). In both intestine and feces, ΔHoc‐PNJ‐6 exhibited lower retention than wild‐type φPNJ‐6 at all time points (Figure 6A−C). This difference was particularly pronounced at 30 h, where retention of ΔHoc‐PNJ‐6 in the cecum and feces was one order of magnitude lower than wild‐type φPNJ‐6 (Figure 6A,C), and in the colon, retention was two orders of magnitude lower (Figure 6B). In contrast, Hoc^Δ29−33^‐PNJ‐6 showed retention in the cecum, colon, and feces approximately one order of magnitude higher than that of wild‐type φPNJ‐6 (Figure 6A−C). These results suggest that Hoc^Δ29−33^‐PNJ‐6 has stronger adhesion and stays longer in the intestine. To visually confirm these observations, we performed indirect immunofluorescence on intestinal sections. Compared with wild‐type φPNJ‐6 (Figure 6D‐i), the Hoc^Δ29−33^‐PNJ‐6 group displayed stronger fluorescence intensity (Figure 6D‐ii), while the ΔHoc‐PNJ‐6 group showed weaker fluorescence (Figure 6D‐iii). The intestinal mucosal layer showed no detectable fluorescence signal in the PBS‐treated control group (Figure 6D‐iv), indicating the absence of phage adhesion. These findings were validated by quantitative analysis of fluorescence intensity (Supporting Information 2: Figure S4). These results are consistent with our previous cell‐based experiments and protein structural analyses, indicating that deletion of Hoc reduces intestinal adhesion, whereas structural changes in Hoc^Δ29−33^ enhance adhesion.

Figure 6Enhanced gut adhesion and protective efficacy of engineered phage in mice. (A–C) Analysis of phage adhesion capacity in the murine intestinal tract, showing phage titers in the cecal contents (A), colonic contents (B), and feces (C) at various time points postadministration. (D) Immunofluorescence imaging of phage adhesion to mouse colon sections. The arrow indicates the colon lumen. Red: MUC2; Blue: DAPI (nuclei); Green: phage particles. Scale bar: 50 µm. (D1) Colon section from φPNJ‐6‐treated mouse, showing wild‐type level phage adhesion. (D2) Colon section from Hoc^Δ29−33^‐PNJ‐6‐treated mouse, showing enhanced phage adhesion. (D3) Colon section from ΔHoc‐PNJ‐6‐treated mouse, showing reduced phage adhesion. (D4) Colon section from PBS‐treated mouse. (E–H) Bacterial loads of *E. coli* CVCC232 in the prevention model, quantified in caecum mucus (E), caecum contents (F), colon mucus (G), and colon contents (H). (I–L) Phage persistence in the prevention model, showing phage titers in caecum mucus (I), caecum contents (J), colon mucus (K), and colon contents (L).  ^∗^ *p* < 0.05;  ^∗∗^ *p* < 0.01;  ^∗∗∗^ *p* < 0.001;  ^∗∗∗∗^ *p* < 0.0001. ns stands for not significant, indicating that the difference between the compared groups was not statistically significant (*p* > 0.05).

**Figure:**
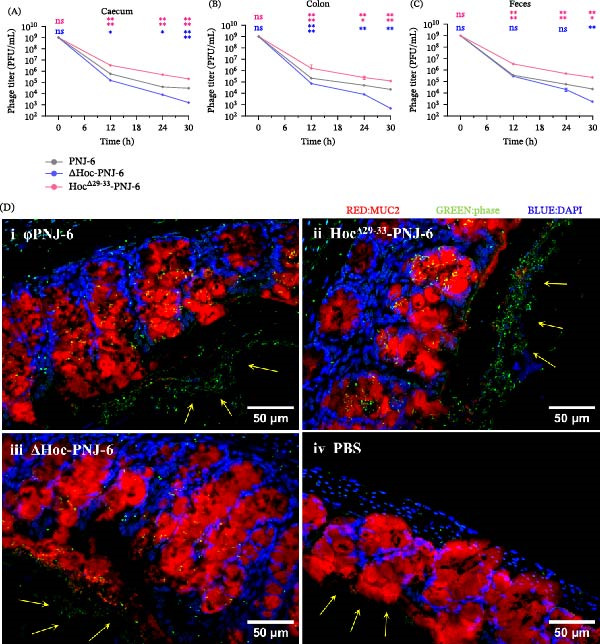

**Figure:**
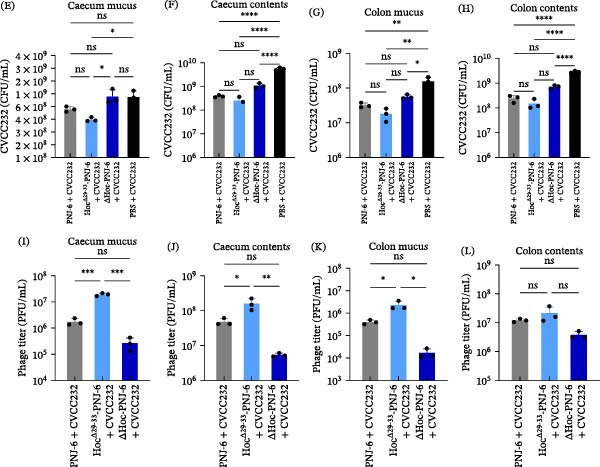

To investigate whether changes in intestinal adhesion affect phage bactericidal activity, we conducted a mouse prophylaxis experiment. Four‐week‐old mice were gavaged with phage, wild‐type φPNJ‐6, or PBS. After 6 h, mice were gavaged with *E. coli* CVCC232, and 12 h postinfection, phage and bacterial loads were measured in the intestinal mucosa and contents. Compared with the φPNJ‐6 group, bacterial loads were higher in both the ΔHoc‐PNJ‐6 and PBS groups but reduced in the Hoc^Δ29−33^‐PNJ‐6 group. This trend was observed in both the mucosal layers of the caecum (Figure 6E,F) and colon (Figure 6G,H) as well as in the intestinal contents. These findings indicate a contrasting trend: deletion of the Hoc protein weakens, whereas the Hoc^Δ29‑33^ mutation enhances the in vivo antibacterial activity of φPNJ‑6. Regarding phage load, the Hoc^Δ29−33^‐PNJ‐6 group exhibited significantly higher titers in the caecum mucosa (Figure 6I), cecal contents (Figure 6J), and colon mucosa (Figure 6K) compared with φPNJ‐6 and ΔHoc‐PNJ‐6, with a similar trend observed in colon contents (Figure 6L). In summary, Hoc^Δ29−33^‐PNJ‐6 demonstrated enhanced intestinal adhesion compared with wild‐type φPNJ‐6, which allows the phage to occupy more intestinal colonization sites, providing greater opportunities to interact with pathogenic bacteria and thereby facilitating the elimination of intestinal pathogens.

## 4. Discussion

Previous studies have shown that phages are more abundant on the mucosal surface than in surrounding nonmucosal environments, where they contribute to the protection of the mucosa against pathogen invasion [28]. This increased abundance is thought to result from adhesion; the reversible binding of Ig‐like domains on the phage capsid to variable glycan residues in mucins allows phage to accumulate in the mucus layer, forming a nonhost‐derived “antimicrobial layer” [23, 29]. These findings highlight an active role for phages in host defense, although the precise mechanisms by which phages regulate mucosal infections remain unclear. Building on this, our team previously identified key glycan‐binding pockets and amino acid residues in the Hoc protein through point mutation analysis and confirmed that Hoc mediates the adhesion of φPNJ‐6 to the gastrointestinal mucus layer in mice [24]. Here, we conducted a more in‐depth study of the key glycan pockets of phage φPNJ‐6 and found that the lock‐and‐key model is insufficient to explain the interaction between Hoc and mucin MUC2. Our results suggested that structural changes in Hoc can enhance their affinity for MUC2.

For T4 phage editing, Cas12a facilitates homologous recombination with greater efficiency than that of Cas9. Moreover, Cas12a recognizes a distinct PAM sequence (TTTN) and generates staggered DNA ends, features that facilitate precise gene replacement and minimize off‐target effects in complex genomes [30]. Therefore, the Cas12a system was selected for this study. We subsequently employed the CRISPR‐Cas12a system to engineer the genome of φPNJ‐6, generating the mutants ΔHoc‐PNJ‐6 and Hoc^Δ29−33^‐PNJ‐6. The mutant phages ΔHoc‐PNJ‐6 and Hoc^Δ29−33^‐PNJ‐6 showed no significant differences from the wild type in pH sensitivity, optimal MOI, or in vitro lytic activity. However, one‐step growth curve analysis revealed that ΔHoc‐PNJ‐6 exhibited both a prolonged latent period and a delayed entry into the logarithmic growth phase. Hoc is a high‐copy‐number, nonessential protein located on the bacteriophage T4 capsid [19]. It may assist in the initial attachment of the phage to the host cell surface, thereby facilitating subsequent receptor recognition and binding by the tail fibers [31]. We therefore speculate that Hoc coordinates early interactions with the host during the initial stages of infection. Consequently, its complete absence could induce a transient delay in the initial adsorption step. This delay would then be reflected as a prolonged latent period in the one‐step growth curve.

Cell adhesion assays showed that ΔHoc‐PNJ‐6 exhibited reduced adhesion, indicating that Hoc is a key protein mediating φPNJ‐6 colonization of the mucosal layer, consistent with our previous findings. Unexpectedly, Hoc^Δ29−33^‐PNJ‐6, which lacks the key MUC2‐binding motif of Hoc, displayed increased adhesion. In vivo adhesion assays further confirmed this observation. These results indicated that the key binding sites of φPNJ‐6 identified in our previous studies are insufficient to fully explain the interaction between Hoc and MUC2. Partial deletion of amino acid sequences often leads to protein structural alterations. For example, key mutations in the receptor‐binding domain (RBD) of the Omicron BA.4/BA.5 subvariants disrupt the binding of multiple neutralizing antibodies while maintaining or even enhancing affinity for the ACE2 receptor through compensatory interactions [32]. Similarly, bioinformatic analyses revealed that Hoc^Δ29−33^ adopts a structurally altered conformation compared with Hoc. These changes result in an increased number of predicted protein pockets in Hoc^Δ29−33^. Protein pockets are potential surface cavities capable of binding small‐molecule ligands, and changes in their number or position can affect protein‐ligand affinity [32–35]. This suggests that newly formed pockets in Hoc^Δ29−33^ may influence their interactions with various proteins or small‐molecule ligands. As our previous work demonstrated that fucose residues constitute the interaction sites between MUC2 and Hoc [24], we first investigated the interaction between Hoc^Δ29−33^ and fucose. Fucose‐binding site prediction showed that Hoc^Δ29−33^ possesses one fewer fucose‐binding pocket than Hoc and that these pockets do not overlap in position. However, not all predicted pockets are functionally engaged under physiological conditions, so the reduction in the number of predicted glycan‐binding pockets in Hoc^Δ29−33^ did not attract our primary attention. Previous studies have reported that even a single mutation in Hoc can affect phage binding to glycosylated mucin glycans [36]. Notably, the number of predicted fucose‐binding residues in Hoc^Δ29−33^ was approximately twice that of Hoc, and SPR assays further confirmed that Hoc^Δ29−33^ exhibits a higher affinity for fucose. These findings indicate that deletion of amino acids 29–33 in Hoc induces structural alterations that consequently enhance the affinity of Hoc^Δ29−33^ for fucose. In other words, the enhanced adhesion capacity of Hoc^Δ29−33^‐PNJ‐6 is associated with the higher fucose affinity of Hoc^Δ29−33^. However, whether these structural changes also affect the binding capacity of Hoc^Δ29−33^ to other proteins or small‐molecule ligands remains unclear.

Phage adhesion capacity is fundamental for their establishment in the gut and for sustaining homeostatic and immunomodulatory functions. Phage adhered to the mucus layer forms a dynamic “antimicrobial layer” between intestinal epithelial cells and the microbiota, continuously lysing bacteria in close proximity to the epithelium [37, 38]. The enhanced adhesion capacity of Hoc^Δ29−33^‐PNJ‐6 allows it to contact and lyse a greater number of pathogenic bacteria. In the mouse prophylaxis model, mice treated with Hoc^Δ29−33^‐PNJ‐6 showed a trend toward reduced bacterial load in both intestinal contents and the mucus layer compared to that in other groups, although this difference was not statistically significant. Hoc is a nonessential protein during the phage replication cycle. However, previous studies have reported that the combined deletion of the phage head protein gp24 and the nonessential protein Soc can affect phage adaptability [39]. Similarly, knockdown of the nonessential protein PhuZ using programmable antisense oligonucleotides caused phage φKZ to exhibit reduced plaque formation and delayed host lysis [40]. These phenomena indicate that alterations in nonessential proteins may affect phage adaptability, which may explain why the protective capacity of Hoc^Δ29−33^‐PNJ‐6 did not show a significant enhancement.

In summary, through gene knockout approaches, we demonstrated that the interaction between the head capsid protein Hoc of *E. coli* phage φPNJ‐6 and MUC2 does not conform to a rigid point‐to‐point “lock‐and‐key” binding mode. We engineered an adhesion‐enhanced phage variant, Hoc^Δ29−33^‐PNJ‐6. Benefiting from the enhanced fucose affinity of Hoc^Δ29−33^, Hoc^Δ29−33^‐PNJ‐6 exhibits a stronger intestinal colonization capacity. This property may enable the phage to adhere to and target intestinal pathogens more effectively. Deletions in other regions of the Hoc protein, such as varying lengths of the N‐ or C‐terminus, could yield distinct phenotypes. The phenotypic outcome would likely depend on whether the deleted region is directly involved in interacting with mucus components. Therefore, systematically investigating how deletions in different Hoc domains affect adhesion is both a limitation of this work and an important direction for future research. Simultaneously, the potential impact of nonessential proteins on phage adaptability should be carefully considered during phage engineering. Our findings expand the current understanding of Hoc‐mucin interactions and provide a potential direction for the design of long‐term mucosa‐resident therapeutic phage formulations. Consequently, this strategy offers a promising, precise biocontrol tool against bacterial diarrhea in livestock and poultry. Its application could reduce the reliance on antibiotics in animal farming.

## 5. Conclusion

We constructed the phage variant Hoc^Δ29−33^‐PNJ‐6, which enhances adhesion. Bioinformatics analysis revealed that the structure of the Hoc^Δ29−33^ protein in the mutant Hoc^Δ29−33^‐PNJ‐6 had changed, with an increased protein pocket and a two‐fold increase in the binding site for fucose. Consistent with this, the Hoc^Δ29−33^ protein exhibited enhanced fucose affinity. Accordingly, the Hoc^Δ29−33^‐PNJ‐6 variant exhibited a stronger intestinal colonization ability. Furthermore, the impact of nonessential proteins on phage adaptability must be carefully considered in phage engineering. The results demonstrated that the Hoc‐MUC2 interaction does not conform to a simple “lock‐and‐key” model and that artificial modulation of the spatial conformation of Hoc can influence the mucosal adhesion capacity of bacteriophages.

## Author Contributions

Fang Tang designed the experiments. Jianjun Dai provided valuable suggestions for the manuscript. Linlin Ye wrote the manuscript and performed most of the experiments described in the manuscript. Xuhang Wang, Jiaqi Cui, Xinru Chen, Pan Tao, Yuepeng Liu, Yuhan Zhang, and Feng Xue offered help during the experiments.

## Funding

This work was supported by the Outstanding Youth Fund of Jiangsu Provincial Natural Science Foundation (Grant BK20240090) and the National Key Research and Development Program of China (Grant 2023YFD1800300).

## Disclosure

All authors have read and approved the final version of the manuscript. The funders had no role in study design, data collection and analysis, decision to publish, or preparation of the manuscript. Statistical analysis was performed using GraphPad Prism software (GraphPad Software, La Jolla, CA, USA). Error bars represent 95% confidence intervals, with the midline indicating the mean ± standard deviation for histograms and line graphs. Data from temperature sensitivity, pH sensitivity, cell adhesion, and animal experiments were analyzed by two‐way ANOVA. One‐step growth curve and phage lysis curve data were evaluated using one‐way ANOVA. A *p* value of less than 0.05 was considered statistically significant, as indicated by asterisks (ns, *p* > 0.05;  ^∗^ *p* < 0.05;  ^∗∗^ *p* < 0.01;  ^∗∗∗^ *p* < 0.001; and  ^∗∗∗∗^ *p* < 0.0001).

## Ethics Statement

All the mice used in this study were purchased from the Comparative Medicine Center of Yangzhou University. All experiments involving mice were conducted in accordance with the Guide for the Care and Use of Laboratory Animals and were approved by the Institutional Animal Care and Use Committee of the Experimental Animal Center (Approval Number PZ2020065).

## Conflicts of Interest

The authors declare no conflicts of interest.

## Supporting Information

Additional supporting information can be found online in the Supporting Information section.

## Supporting information


**Supporting Information 1** Table S1: Nucleotide and amino acid sequences in this study.

**Supporting Information 2** Figure S1: Identification of genetic stability of modified phage gene editing sites. Figure S2: Comparison of phage lytic activity under acidic conditions. Figure S3: Phage titers in the cell‐based prevention assay. Figure S4: Quantitative analysis of phage fluorescence intensity in intestinal immunofluorescence sections.

## Data Availability

The genome sequence of phage φPNJ‐6 has been deposited in NCBI GenBank under the accession code OQ076693.1. Sequences for Hoc domains, Hoc, and Hoc^Δ29−33^ are provided in Supporting Information 1: Table S1. The source data are available with this paper.
