# Supplementary material for: Structural Remodeling of Phage φPNJ‐6 Hoc Promotes Adhesion to the Intestinal Epithelium
Source: Transbound Emerg Dis. 2026 Jun 9;2026:2424208. doi: 10.1155/tbed/2424208 (PMC13248984; doi:10.1155/tbed/2424208)
Supplement: Supplementary file 2 — Supporting Information 2 Figure S1: Identification of genetic stability of modified phage gene editing sites. Figure S2: Comparison of phage lytic activity under acidic conditions. Figure S3: Phage titers in the cell‐based prevention assay. Figure S4: Quantitative analysis of phage fluorescence intensity in intestinal immunofluorescence sections. [file TBED-2026-2424208-s001.docx]

**Supplementary information for**

**Structural remodeling of phage φPNJ-6 Hoc promotes adhesion to the intestinal epithelium**

Linlin Ye^1^, Xuhang Wang^1^, Jiaqi Cui^1^, Xinru Chen^1^, Pan Tao^4^, Yuepeng Liu^3^, Yuhan Zhang^1^, Feng Xue^1, 2^, Jianjun Dai^1, 2^, Fang Tang^1, 2, *^

* Corresponding authors: Fang Tang

Email: tfalice@126.com (FT)

**Supplementary Figure S1**

**

**

**Fig. S1 Identification of genetic stability of modified phage gene editing sites.** (A) PCR electrophoresis map; (B) sequencing peak map comparison. M: 2000 DNA Marker; 1: PNJ-6; 2: Hoc^Δ29-33^-PNJ-6; 3: ΔHoc-PNJ-6.

**Supplementary Figure S2**

**

**

**Fig. S2 Comparison of phage lytic activity under acidic conditions.** Lysis curves of the PNJ-6, ΔHoc-PNJ-6 and Hoc^Δ29-33^-PNJ-6 following treatment with SM buffer at pH (A) 2.0, (B) 4.0, and (C) 6.0. ns, *p*>0.05.

**Supplementary Figure S3**





**Fig. S3 Phage titers in the cell-based prevention assay.** Titers of phage present in the planktonic phase of the culture supernatant and those adherent to cells were quantified at 3 h (i), 6 h (ii), and 12 h (iii) post-co-culture with *E. coli* CVCC232. ns, *p*>0.05.

**Supplementary Figure S4**





**Fig. S4 Quantitative analysis of phage fluorescence intensity in intestinal immunofluorescence sections.** Fluorescence intensity value= IntDen/Area. Statistical analysis was performed using One-way ANOVA analysis, ****, *p*< 0.0001.
